# Pulmonary metastasectomy for esophageal basaloid squamous cell carcinoma component at 66 months after esophagectomy

**DOI:** 10.1186/s40792-020-00957-z

**Published:** 2020-08-05

**Authors:** Kumiko Sekiguchi, Takeshi Matsutani, Tsutomu Nomura, Nobutoshi Hagiwara, Akihisa Matsuda, Hidetsugu Hanawa, Keisuke Mishima, Nobuhiko Taniai, Ryuji Ohashi, Hiroshi Yoshida

**Affiliations:** 1 Department of Gastrointestinal Hepato-Biliary-Pancreatic Surgery, Nippon Medical School, 1-1-5 Sendagi, Bunkyo-ku, Tokyo, 113-8603 Japan; 2 Department of Digestive Surgery, Nippon Medical School Musashikosugi Hospital, 1-396 Kosugimachi, Nakahara-ku, Kawasaki-shi, Kanagawa 211-8533 Japan; 3 Department of Integrated Diagnostic Pathology, Nippon Medical School, 1-1-5 Sendagi, Bunkyo-ku, Tokyo, 113-8603 Japan

**Keywords:** Esophageal basaloid squamous cell carcinoma, Lung metastasis, Pulmonary resection

## Abstract

**Background:**

Solitary pulmonary metastasis from esophageal basaloid squamous cell carcinoma (BSCC) components is an extremely rare recurrence of esophageal squamous cell carcinoma (SCC).

**Case presentation:**

A 68-year-old Japanese woman was found to have a suspected malignant mass, approximately 2 cm in diameter, in her left lower pulmonary lobe, at 66 months after undergoing a curative esophagectomy with three-field lymph node dissection for esophageal SCC with a focal basaloid component. After a CT-guided biopsy, pathological examination indicated a metastasis from esophageal BSCC components. She underwent a thoracoscopic partial resection of the left lower pulmonary lobe for the solitary pulmonary metastasis. The pathohistology of the resected specimen led to diagnosis of metastatic esophageal BSCC, which showed immunohistochemical findings similar to those of the primary esophageal carcinoma. The patient received two courses of adjuvant chemotherapy (5-fluorouracil, docetaxel plus nedaplatin) and recovered to resume a normal life with maintenance therapy. However, multiple lung and brain metastases were diagnosed at 2 years after the pulmonary metastasectomy. She survived 5 years and 6 months after the pulmonary metastasectomy, but died at 10 years and 6 months after her initial esophagectomy.

**Conclusion:**

This was a rare surgical resected case of solitary pulmonary metastasis from esophageal BSCC components.

## Introduction

Resection of solitary metastases to the lung from various primary malignancies, such as colorectal cancer and head and neck cancer, in the absence of other metastases, is reportedly becoming the standard therapy [1, 2]. Although esophageal squamous cell carcinoma (SCC) frequently metastasizes to the lung, pulmonary metastasectomy is not generally considered as an initial standard therapy for recurrence of esophageal SCCs. However, several studies have examined the role of pulmonary metastasectomy for patients with esophageal SCCs [3–10]. In this report, we described a successful pulmonary metastasectomy for a solitary pulmonary metastasis from esophageal SCC that was primarily composed of basaloid squamous cell carcinoma (BSCC) components.

## Case report

**Fig. 1:**
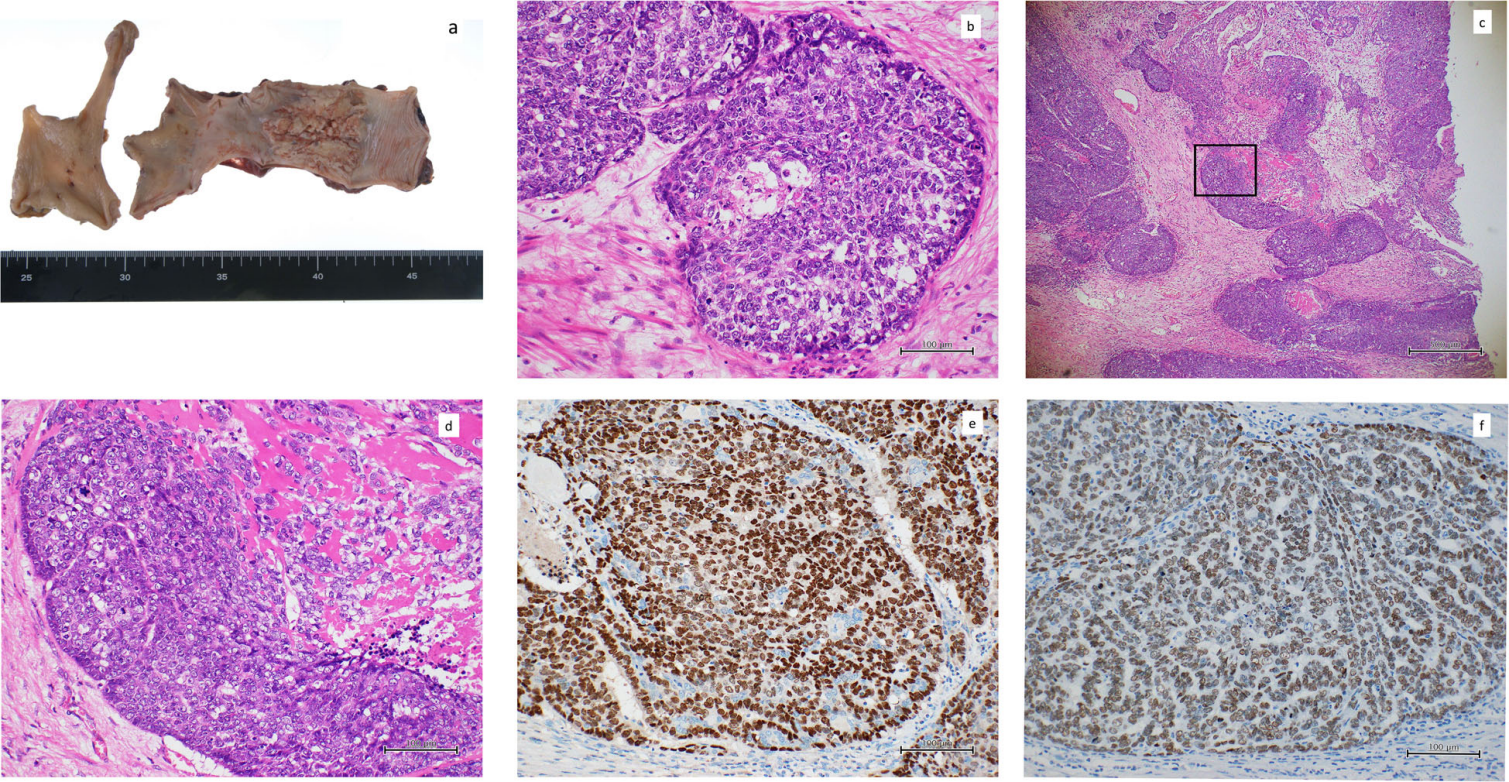
**a** Macroscopic appearance of the opened esophagus and stomach shows an ulcerative and infiltrative type tumor in the middle thoracic esophagus. Histopathologic examination of the resected specimen shows moderately differentiated squamous cell carcinoma (**b** hematoxylin and eosin stain, ×100), with a focal basaloid component (square, **c** × 4 and **d** × 100). Immunohistochemical examinations show that the primary tumor cells are positive for **e** p63and **f** p40 (× 100)

**Fig. 2:**
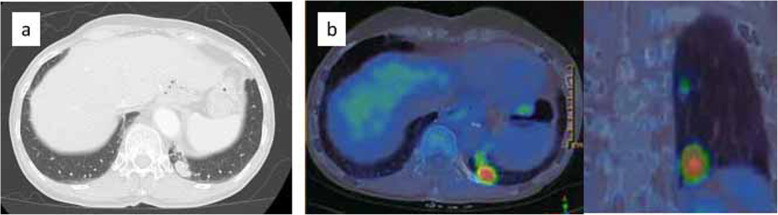
**a** Chest computed tomography scan shows a solitary oval pulmonary lesion, approximately 20 × 11 mm, in the left lung. **b** Fluorodeoxyglucose positron emission tomography-computed tomography confirms the presence of one lesion in the lung and the absence of metastases in the lymph nodes or extrapulmonary sites

**Fig. 3:**
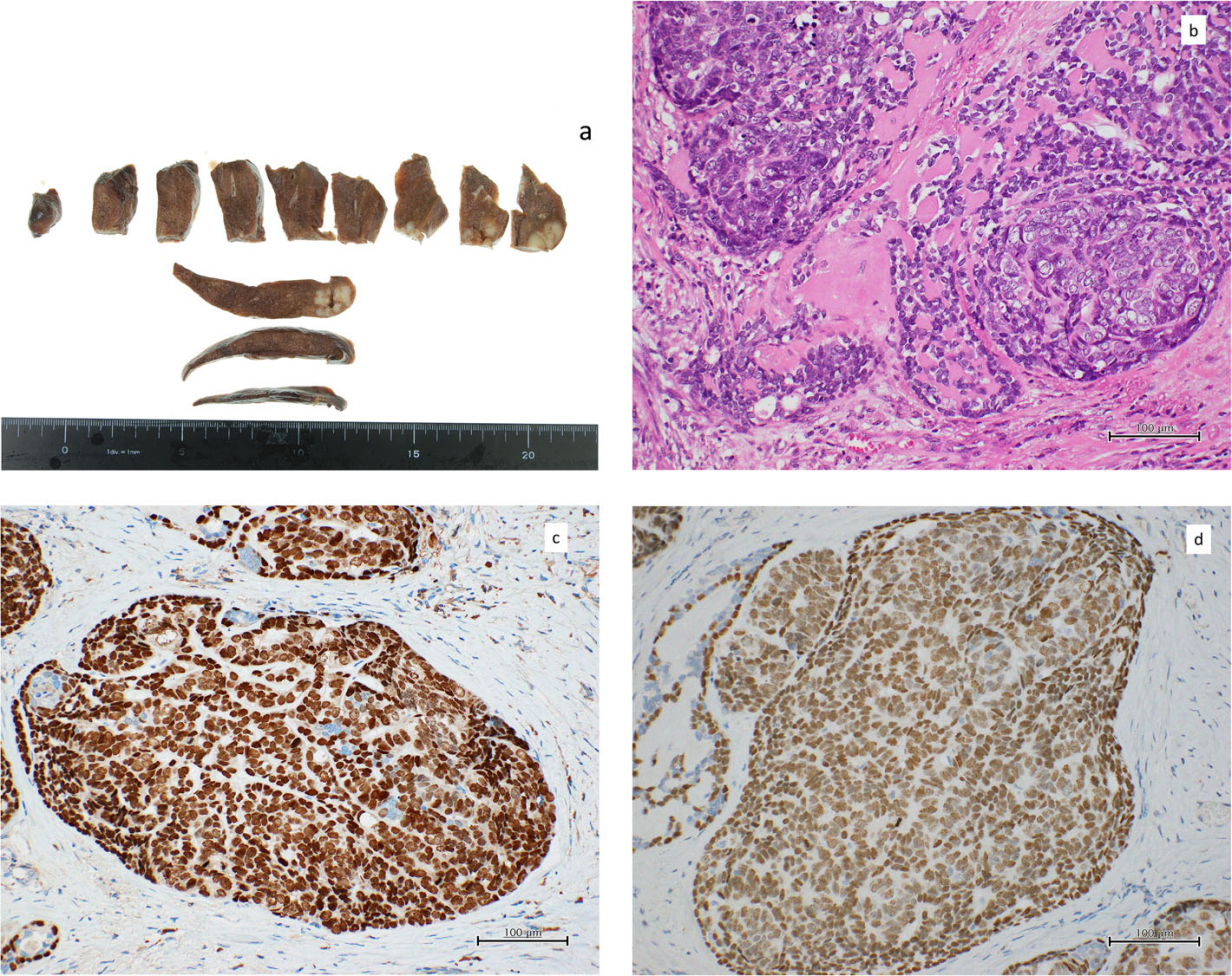
**a** Macroscopic observation of the resected left lung specimen shows a white nodule with irregular borders. **b** Histological evaluation of the pulmonary lesion, leading to diagnosis of metastasis of basaloid squamous cell carcinoma of the esophagus with deposition of hyaline-like material (hematoxylin and eosin stain, × 100). Immunohistochemical examinations show that the metastatic tumor cells are positive for **c** p63 and **d** p40 (× 100)

A 68-year-old Japanese woman presented with a 2-cm mass in her lower left lung. Sixty-six months previously, she had undergone a curative esophagectomy via a right thoracotomy with three-field lymph node dissection for advanced esophageal cancer. A gastric tube was created during an open laparotomy and used as an esophageal substitute through the postmediastinal route. Pathological examination of the all-segmented tumor specimens revealed the proliferation of large squamoid cells with enlarged nuclei forming nests with a central necrosis, infiltrating throughout the esophageal wall (Fig. 1a–d). Some of the tumor nests exhibited ambiguous nuclear palisading arrangement in the peripheral region, in which deposition of hyaline-like materials was occasionally identified. These histological features were diagnostic of moderately differentiated SCC with a focal basaloid component. In accordance with the Union for International Cancer Control TNM staging system (7th edition), the tumor was classified as pT3N0M0, pStage IIA. Immunohistochemical examinations showed that the tumor cells were positive for p63 and p40 (Fig. 1e, f) and negative for SP-A, TTF-1, chromogranin A, synaptophysin, S-100, and calponin. However, lymphatic and venous invasions were detected. She then received adjuvant chemotherapies combined with docetaxel (DOC; 40 mg/body) plus nedaplatin (CDGP; 40 mg/body). Routine follow-up chest x-rays and computed tomography (CT) scans showed no masses in the lungs or other abnormalities for more than 5 years after adjuvant chemotherapies. She never complained of gastrointestinal or pulmonary symptoms. However, at 66 months after the surgery, a chest CT detected a solitary, oval-shaped, 20 × 11-mm lesion in the left lung (Fig. 2a). An ^18^F-fluorodeoxyglucose positron-emission tomography (FDG-PET)-CT scan revealed abnormal FDG uptake in the left lung, with a standardized uptake value of 9.58 (Fig. 2b). Pathological findings from a CT-guided biopsy specimen indicated the presence of BSCC components. As systemic examinations revealed no other metastases, we decided to remove it surgically and performed a partial resection of the lower left lobe via thoracoscopy. No pleural dissemination was observed intraoperatively. The postoperative course was uneventful. Histological features of the metastatic tumor were similar to the basaloid component of the primary tumor in the esophagus (Fig. 3a, b). Nests of polygonal cells of various sizes in an alveolar configuration showed a massively expansive-infiltrative growth pattern in a sheet formation with comedo necrosis. The presence of scant cytoplasm, round to oval nuclei, a high nuclear to cytoplasmic ratio, relativistic nuclear palisading arrangement in the marginal region of the tumor, and deposition of hyaline-like materials was compatible with BSCC of the esophagus. We also observed characteristic PAS-positive hyaline material in part of the alveolar interstitium. There was no existing bronchial/alveolar cavity in the metastatic cancer foci or alveolar replacement growth around the peripheral lung tumor. The immunohistochemical examination indicated that the tumor had metastasized from the primary esophageal SCC, because its cells were positive for p63 and p40 (Fig. 3c, d) and negative for SP-A and TTF-1. Finally, we diagnosed the tumor as a metastasis from BSCC components of the esophagus. The patient was discharged from our hospital on postoperative day 9 and then received two courses of adjuvant chemotherapy of 5-fluorouracil (5-FU) and CDGP plus DOC (5-FU 450 mg/m^2^: Days 1–5, CDGP 20 mg/body: Days 1–5, DOC 80 mg/body: Day 1). Although the patient did well initially without any recurrence after the pulmonary metastasectomy, multiple lung and brain metastases were diagnosed 2 years later on follow-up chest/brain CT examinations. The patient died for 10 years and 6 months after the initial esophagectomy.

## Discussion

Metastases from esophageal SCC are most commonly diagnosed in lymph nodes (45%) followed by liver (35%), lung (20%), bone (10%), adrenal gland (5%), and peritoneum (2%) [11–14]. Although the lung is the third most common site for hematogenous metastasis from esophageal SCC, seven studies of pulmonary metastasectomy as a therapeutic modality are available [3–9]. The indications for these procedures and their prognostic factors are not yet well characterized. Generally, the survival of patients with hematogenous metastasis was significantly worse than that of patients with locoregional recurrence. Kato et al. [15] reported that the 3-year survival rate for esophageal cancer patients with hematogenous metastasis is only 0.3%. Ichikawa et al. [4] retrospectively found that the 5-year survival rate of 23 patients who underwent pulmonary metastasectomy for esophageal SCC was relatively good (43.5 %), and that initial recurrence in the form of extrapulmonary metastasis was an unfavorable prognostic factor. Chen et al. [3] demonstrated that patients with solitary pulmonary metastases could prognostically benefit from pulmonary metastasectomy. Both authors suggested pulmonary metastasectomy for esophageal carcinoma should be considered in selected patients who had few solid lesions without extrapulmonary metastasis. However, small numbers of patients in both studies limited their statistical analyses. A larger prospective study is required to confirm the efficacy of pulmonary metastasectomy for esophageal carcinoma metastases.

Although SCC is the predominant histological type among esophageal malignancies in Asian countries, BSCC of the esophagus is an uncommon SCC variant; it comprises approximately 1.5% of esophageal carcinomas in Japan [16]. Whether esophageal BSCC is oncologically similar to SCC is also unclear. Despite curative resection or adjuvant therapy, advanced BSCC usually has a fatal outcome, because of its high proliferative activity, its rapid and widespread lymphogenous and hematogenous metastases, and its highly malignant potential [17, 18]. The most widely accepted histogenetic hypothesis for composite tumors of the esophagus is the neoplastic transformation of primitive totipotent cells in the basal squamous mucosa, leading to heterogeneous differentiation within a primary tumor [19, 20]. In the present case, the primary component of the lung metastasis was BSCC, which characteristically shows aggressive biological behavior. The immunohistochemical examination indicated that both primary and metastatic cells were positive for p63 and p40. The p40 antibody recognizes ΔNP63, an isoform of p63, and ΔNP63 has been suggested to be highly specific for squamous/basal cells. However, proving it to be a composite tumor of the esophagus could be inconclusive, because ordinary esophageal SCC sometimes shows partial glandular differentiation.

Although primary BSCC of the lung is rare, the distinction between primary and metastatic BSCC is necessary in our case. In general, clinical examinations such as CT, PET-CT, and tumor markers cannot solely help to differentiate primary from metastatic lung cancer. The definitive diagnosis should be made taking into account all the test results, including clinical, morphological, immunohistochemical, and molecular biological findings. In our case, the lung tumor cells had BSCC features, which were also identified within some of the tumor nests in the esophagus. In addition, the lung tumor was relatively demarcated, and the surrounding pulmonary parenchyma showed no atypia, all of which are highly supportive of metastatic cancer. Unfortunately, there are no organ-specific immunohistochemical markers for BSCC to differentiate metastatic from primary BSCC in the lung. Although there is no definitive evidence such as molecular test results, we collectively judged that the lung tumor in our case was metastasis from the esophageal SCC.

Patients with pulmonary metastasis from esophageal carcinoma are usually in poor condition at presentation and may receive only palliative treatment. Radical treatments are selected for patients in relatively good general condition with localized metastasis. Komatsu et al. [6] demonstrated that if the patients who have received chemotherapy before pulmonary metastasectomy were poor responders to chemotherapy, they may have missed the best timing for surgery. Surgery might be considered when complete resection of metastatic tumors is possible. However, in many cases, hematogenous recurrence, such as pulmonary metastases, cannot be completely treated by surgical resection because of the high likelihood of impending tumor development or coexistence of metastases to other organs. In principle, chemoradiotherapy, regarded as a locoregional treatment, should not be used as a radical treatment for patients with distant organ metastasis, although systemic chemotherapy is still widely accepted as a standard treatment for such patients. Esophageal SCC is generally responsive to cisplatin (CDDP)-based combination chemotherapy [12, 21]. CDGP, a second-generation platinum complex, is a characteristic property of being approximately 10 times more soluble in water than CDDP, and is therefore considered to stronger activity against solid tumors and less nephrotoxicity and gastrointestinal toxicity than CDDP [21].

A literature and database research found three patients treated with pulmonary metastasectomy for solitary BSCC metastasis [22–24]; their clinicopathological data, along with our patient’s data, are summarized in Table 1. These reported three patients received no adjuvant chemotherapy and survived without postoperative recurrence. However, our patient received adjuvant chemotherapy because metastatic BSCC is an aggressive SCC subtype.

**Table 1:** Reported patients who received pulmonary metastasectomies for basaloid squamous cell carcinoma

1st author, year, reference	Age/sex	Stage (path)	Primary tumor path	DFI, mo	Tumor size, mm	Metastasectomy	DFS, mo	OS, mo	Outcome
Takemura, 2012 [20]	63/F	IIB, pT1N1M0	BSCC	24	10	Wedge resection	84	84	Alive
Shibasaki, 2012 [19]	67/F	IIA, pT3N0M0	BSCC	24	10	Partial resection	17	17	Alive
Kosaka, 2014 [18]	78/F	IIIB, pT3N2M0	BSCC	44	16	Partial resection	12	12	Alive
Present case	68/F	IIA, pT3N0M0	SCC	66	20	Partial resection	24	60	Dead

All patients in these reports had unilateral procedures for a single metastasis each, and their metastases were pathologically found to be BSCC.

*BSCC* basaloid squamous cell carcinoma, *DFI* disease-free interval of initial esophagectomy, *DFS* disease-free survival of lung metastasectomy, *mo* months, *OS* overall survival of lung metastasectomy, *Stage (path)* 7th edition International Cancer Control TNM stage (pathology), *SCC* squamous cell carcinoma

## Conclusion

We treated a rare case of a resected solitary pulmonary metastasis of BSCC components from an esophageal SCC primary. As few cases of resected pulmonary metastases from BSCC of the esophagus are reported, no standard treatment strategy has been established. Additional reports are required to determine the optimal treatment.

## Data Availability

The data are not available for public access due to patient privacy concerns but are available from the corresponding author on reasonable request.

## Abbreviations

SCC: Squamous cell carcinoma
BSCC: Basaloid squamous cell carcinoma
DOC: Docetaxel
CDGP: Nedaplatin
CT: Computed tomography
FDG-PET: ^18^F-fluorodeoxyglucose positron-emission tomography
5-FU: 5-Fluorouracil
CDDP: Cisplatin
